# Correction: MaskDGNets: Masked-attention guided dynamic graph aggregation network for event extraction

**DOI:** 10.1371/journal.pone.0320695

**Published:** 2025-03-21

**Authors:** Guangwei Zhang, Fei Xie, Lei Yu

There is an error in affiliation 1 for author Guangwei Zhang. The correct affiliation 1 is: Beihang University, Beijing, China.
