# Supplementary material for: MaskDGNets: Masked-attention guided dynamic graph aggregation network for event extraction
Source: PLoS One. 2024 Nov 15;19(11):e0306673. doi: 10.1371/journal.pone.0306673 (PMC11567535; doi:10.1371/journal.pone.0306673)
Supplement: Attachment — Submitted filename: manuscript for publication.docx [file pone.0306673.s001.docx]

Antibacterial activity of *Cassia tora* leaf extract against some bacteria isolates from Urinary Tract Infected patients attending Aminu Kano Teaching Hospital, Kano

Abdulrasheed usman ^1*^, Auwal Bello Muhammed ^2^ , Rahinatu Sharfadi ^3^

**1** Department of Medical Laboratory Science, Faculty of Allied Health Sciences Bayero University, Kano, Nigeria

**2** Department of Medical Laboratory Science, Faculty of Allied Health Sciences Bayero University, Kano, Nigeria

**3** Department of Medical Laboratory Science, Faculty of Allied Health Sciences Bayero University, Kano, Nigeria

*correspondingauthor: uabdulrasheed10@gmail.com

# Abstract

Urinary tract infection (UTI) usually refers to the presence of bacteria (> 10ˆ5 bacteria per ml of urine) in the urinary tract together with symptoms, and sometimes signs of inflammation. It is characterized by frequency of dysuria, pyuria, fever, suprapubic pain, and haematuria. The study aimed to determine the antibacterial activities of *Cassia tora* leaves extracts against some bacteria isolated from UTI patients attending Aminu Kano Teaching hospital Kano (AKTH). Collection of plant materials was done by selecting Mature, fresh, and healthy samples of *C. tora* within Kano metropolis, it was identified at the Department of Plant Biology Bayero University, Kano. The plant extraction was carried out by soxhlet extraction method and methanol as the solvent. Consecutive urine specimens from patients with UTI at Aminu Kano Teaching Hospital Kano, Nigeria were collected, processed, and examine microscopically. Bacterial pathogens were isolated by culture, identified by biochemical test and the isolates were subjected to antimicrobial susceptibility testing against *Cassia tora* leaves extract. A total of 161 subjects presented with UTI were enrolled in the study. Out of the total samples under the study, 32 samples yielded positive cultures, while 129 have shown no growth. The prevalence rate of 19.9% was recorded in this study. The most implicating organisms causing urinary tract infections in the study were *Escherichia coli,* they were responsible for 41.0% of the cases of UTI. This was followed by other species. The bacterial isolates were screened for antibacterial activities using *Cassia tora* leaf extract in varying concentration and a conventional antibiotic (ciprofloxacin 5ug) was used as a control. The clinical isolates showed relatively high resistance to the plant extract (*Cassia tora*) at the different concentrations with 0 mm zone of inhibitions. The study shows that not all *Cassia tora* leaves extract possess significant antibacterial activity base on the results generated. Further research should be carried out to quantify, compare and contrast between poly phenolic compounds from different geographical distribution and to evaluate their similarities and difference in antimicrobial activities.

Keywords: Anti-microbial activities, plant extraction method, Conventional antibiotic, Pyogene, Resistance, Clinical isolates, Minimum inhibitory concentration, Data representation

# Introduction

Urinary tract infection (UTI) usually refers to the presence of bacteria (> 105 bacteria per ml of urine) in the urinary tract together with symptoms, and sometimes signs, of inflammation [1]. It is characterized by frequency of micturation, dysuria, pyuria, nucturia, fever, occasional suprapubic pain, and haematuria [2-3]. UTI is one of the most commonly occurring bacterial infections among men and women [4-5]. The highest incidence mostly occurs in healthy young women who present with symptoms of acute uncomplicated bacterial cystitis or pyelonephritis [6-7]. UTI is mostly caused by gram-negative aerobic bacilli found in the gastrointestinal tract known as Enterobacteriacae [8]. Included in this family are the *Escherichia coli, Klebsiella, Enterobacter, Citrobacter, Proteus specie* and *Serratia species* [9-10]. Other common pathogens include *Staphylococcus epidermidis,* *Staphylococcus saprophyticus* and *Entercoccus species* which presumably result in UTI following colonization of the vigina or peri anal skin [11, 12, 13]. Less common organisms such as Gardrenella vaginalis, Mycoplasma specie and Ureaplasma urealyticum may infect patients with intermittent or indwelling catheters [14]. Identification of the causative organisms and of their sensitivity to drugs is important because of the range of the organisms and the prevalence of resistant strains [15]. Therapeutic decisions should therefore be based on accurate and up-to-date antibacterial susceptibility [16, 17, 18].

*Cassia tora* (*C. tora*) (sub-family: Caesalpinioideae; Family: Leguminosae/Fabaceae) is a small shrub that grows up in warm moist soil throughout the tropical parts of Asian and African countries. It is known by different names in different places, in Nigeria it is known as Tafasa by the hausa tribe, asuwon by the yoruba and nsigbu-muo by the igbo likewise other names include Foetid *Sennatora*, *Sickle Senna, Wild Senna, Sickle Pod, Coffee Pod,* Tovara, Chakvad, Ringworm Plant. *C. tora* has 10 cm long pinnate leaves, each leaf has three pairs of leaflets that are opposite, ovate, oblong, and oblique at the base. The yellow-colored flowers are bearded in the axel of the leaves. The flowers consist of half-inch diameter five petals. The seeds of *C. tora* are rhombohedral and brown in color. The *C. tora* gets flowers in the rainy season and fruits in the winter season. *C. tora* leaves, seeds and roots are utilized as food ingredients for long). It possesses a wide range of pharmacological activities. *C. tora* is a medicinal plant known for its laxative, antihepatotoxic, antimutagenic, and antiperiodic Properties [19]. In Chinese medicine books, different medical usage is depicted for different parts of *C. tora* plant. In traditional Hindu and Chinese Medicine, its usage has been described as an antioxidant, antimicrobial, antihepatotoxic, antidiuretic, antidiarrhoeal and antimutagenic plant. Also, it has been suggested that *C. tora* may lead to an improvement invisual acuity [20, 21, 22, 23]. The Different parts of the plant (leaves, seed, and root) are reputed for their medicinal value. The leaves of *C. tora* are reported to have antirheumatic activity in folklore practice. Decoction of the leaves is used as a laxative [24]. The seeds of *C. tora* have been used in Chinese medicine as aperients, antiasthenic, and diuretic agents. It’s also given to improve visual activity (eye diseases) and to treat liver disorders [25]. In Korea, the hot extract of seeds is taken orally for the protection of the liver, Leaves and seeds are used in the treatment of skin disorders (Ringworm and itch) [26]. Stem bark extract is used for various skin ailments, rheumatic diseases and as a laxative. In Ayurveda, the plant is used in ‘DadrughaniVati’ and ‘PamariTaila’ [27]. It was reported that seeds of *C. tora* have antioxidant activity and contain many active substances including chrysophenol, emodin, and rhein [28]. Many medicinal properties such as, antihepatotoxic, and anti-mutagenic activities have been attributed to this plant.

The World Health Organization (WHO) estimates that about 80% of people living in developing countries rely almost exclusively on traditional medicines for their primary health care needs. The search for secondary metabolites from plants as substitutes for synthetic drugs has received much attention. This may be because medicinal plants have been found to have active therapeutic properties against many diseases with less or no side effects compared to synthetic drugs. Recently, treatment of diseases or infections with different medicinal plants has been a predominant practice among people especially in rural areas [29, 30]. The rural dwellers relied on herbal medicine since they cannot afford orthodox medicine and this practice has been providing the needed cure for their ailments [31, 32]. Plant-based systems continue to play an essential role in healthcare, and their use by different cultures has been extensively documented**.** Phytochemicals have been for age’s rich sources for successful drugs discovery, and still represent an important tool for the identification of the novel drug. Scientific evaluation of medicinal plants provides evidence-based alternative medicines which form the basis of the herbal drug industry and the discovery of drug targets in the pharmaceutical industry**.** The main asset of medicinal plant-based drug discovery is the existence of ethnopharmacological information providing information for compounds therapeutically effective in humans [33, 34]. Given the increasingly difficult problems of microbial resistance to most antibiotics, medicinal plants are now being considered as credible alternatives for the treatment of UTI infections. Therefore, this study was carried out to determine antibacterial activities of extracts obtained from the leaves of *Cassia tora* using methanol as a solvent.

# Materials and Method

## Study area

The study was conducted in Aminu Kano Teaching Hospital (AKTH) microbiology laboratory .The Hospital is situated within Kano metropolis, Kano state. The hospital is a Tertiary Health Institution which serves as a referral center for both private and public health institutions in and around the state. It lies between latitude 11^o^30`N and longitude 8^o^30`E. Kano state borders Katsina to the north-west, Jigawa state to the north-east, Bauchi state to the south-east and Kaduna state to the south-west. The total land area of Kano state is 20,760 km^2^ with a population of 9,383,682 based on the official result 2006 of National Population and Housing Census [35].

## Study design

The study was a cross-sectional and prospective in design.

## Study population

The study population include all patients presented with urinary tract infections attending AKTH.

## Inclusion criteria

Patient presented with urinary tract infections attending AKTH were included

## Exclusion criteria

All Patients presented with non-urinary tract infections attending AKTH

## Sample size

The prevalence was taken as 11.9 % from previous study conducted by [36]. Therefore the sample size of this study was calculated and determines using the formula as follows:

P=11.9 %= 11.9 ÷100 =0.119

n= $\frac{Z^{2}Pq}{d^{2}}$

n = sample size,

Z = statistic for a level of confidence at 95% =1.96,

P = prevalence 0.119 and

d = allowable error of 5% (d = 0.05).

n=$\frac{{1.96}^{2}\times0.119(1-0.119)}{{0.05}^{2}}$=161

n=161

## Sampling technique

Convenience sampling technique

## Ethical approval

Ethical approval to conduct this research was sought from the Health Research Ethics Committee of Aminu Kano Teaching Hospital.

## Informed consent

The purpose and benefits of the study to the subject was explained and permission to interview and collect specimens was sought. They were informed that Participation in the study is totally voluntary. Participants were not forced or persuaded to participate in the study. Even those who initially accepted to participate were at liberty to withdraw in the course of the study, they were informed that the anonymity of the participants was guaranteed and the confidentiality of the information they provide. Also the conduct of the study did not pose any health risk to the participants.

## Data collection

Socio demographic data was collected using a well-structured questionnaire. Detailed information about the study were given to the client and consent to participate was obtained before patient sample were collected and data taken.

## Sample collection

Participants were instructed on how to collect the urine samples in a sterile wide mouth, screwed cap 50ml container which was stored in a cooler with ice and taken to the laboratory.

## Sample processing

The collected urine samples were processed immediately upon arrival at the Microbiology Laboratory Aminu Kano Teaching Hospital, Kano.

## Microscopic examination

Microscopic examination was carried out on the fresh urine samples for pus cells (WBC) count, RBC, Epithelial cells and other parameters using x40 objective lens in the wet preparations.

## Isolation of bacteria from urine

### Urine cultures

Urine specimens from UTI patients were cultured according to the procedure of [37]. This was done by streaking loopfull of the sample, using sterile inoculation wire loop on to the surfaces of CLED medium. The Inoculated on the CLED media was incubated aerobically at 37ºC for 24 hours [37].

### Smear making

The wire loop was sterilized and allowed to cool. The loop was deeped in the appropriate portion of the sample. The picked sample was spread on a clean grease-free slide and a thin smear was made. It was allowed to air dry. It was fixed by passing the slide three times over a Bunsen flame with the smear facing upward. The smear was allowed to cool [37].

### Gram stain

A drop of sterile normal saline was placed on a well labeled clean grease-free glass slide using a sterile inoculating loop. A colony of an overnight culture of the bacterial isolate was emulsified with the sterile normal saline to make a thin smear. The smear was then allowed to air dry and then heat fix. The slide was flooded with crystal violet (primary stain) for 60s after which the stain was rinsed with water. The smear was flooded with Lugol’s iodine (mordant) for 60sec. The smear was then rinsed with water after 30s. The slide was flooded with a decolourizer (acid-alcohol) for few seconds and rinsed off almost immediately with tap water. The counter stain; neutral red was added and left for 60 s before being rinse off. The stained smear was allowed to air dry and was observe under the microscope using x100 oil immersion objective lens of the microscope. A red or pink observed bacteria indicates gram negative bacteria while blue or purple indicates gram positive bacteria [37].

## Biochemical characterization of the isolates

### Motility test

A ring of plasticine was made on a cover slip at the centre of the ring. A drop of peptone water containing the pure culture was dropped at the centre of the ring. A grease free slide was inverted on the ring and quickly turned over, so that the cover slip would be at the uppermost. It was examined under the microscope .

### Indole test

The test organism was inoculated into peptone water overnight. Indole was tested by adding 0.5ml kovac’s reagent and shake. Indole positive formed a red colour layer at the top of the tube when kavac’s reagent is added.

### Citrate utilisation test

The test organisms was inoculated onto a citrate agar slant using a sterile straight wire loop, the slope was streak with a saline suspension of the test organism and then the butt was stab and incubated at 37°C for 18-24hours. Positive citrate test was indicated by a blue colour .

### Urease test

The test organisms was inoculated onto a urea agar slant using a sterile straight wire loop, the slope was streak with a saline suspension of the test organism and then the butt was stab and incubated at 37°C for 18-24hours. Positive urease test was indicated by a bright pink colour.

### Triple sugar iron (TSI)

The test organisms was inoculated onto the TSI agar slant using a sterile straight wire loop, the slope was streak with a heavy inoculum of the test organism and then the butt was stab and incubated aerobically at 37°C for 24hours.

## Preparation of macfarland standard

1% v/v solution of sulphuric acid was prepared by adding 1 ml of concentrated sulphuric acid to 99 ml of water with well mixing. 1% w/v solution of barium chloride was prepared by dissolving 0.5 g of dihydrate barium chloride (BaCl2.2H2O) in 50 ml of distilled water. Then 0.6 ml of the barium chloride solution was added to 99.4 ml of the sulphuric acid solution and mix. A small volume of the turbid solution was transfer to a capped tube or screw-cap bottle of the same type as used for preparing the test and control inoculates. It was stored in a well-sealed container in the dark at room at temperature (20–28 C), before using

### Standardization of bacterial cell suspension according to Mac Farland standard

Fresh and pure bacteria isolates grown in CLED agar was mixed with 10 ml of sterile distilled water and the turbidity of the suspension was compared with that of 0.5 McFarland standard [38].

## Collection of plant material

Mature, fresh, and healthy samples of *C. tora* were collected within Kano metropolis, the plant was identified by the Department of Botany Bayero University, Kano.

## Plant extraction using Soxhlet method

Fresh plant materials (leaves) was rinsed with sterile water; air-dried for a few days and pulverized using a mortar and pestle. The crushed plant material was loaded into the thimble, which was placed inside the Soxhlet extractor. The side arm was lagged with glass wool. The methanol solvent was heated using the isomantle and was allowed to evaporate, moving through the apparatus to the condenser. The condensate then drips into the reservoir containing the thimble. The level of solvent was allowed to reaches the siphon and it was pours back into the flask and the cycle begins again. The process was run for a total of 16 hours [39].

## Sample storage

The methanol extract of *Cassia tora* was stored in a vial, and was kept in a refrigerator until used.

## Preparation of different concentration of the plant extract

The amount of dried metabolites that were obtained from the extracts was dissolved thereby making extracts of different concentrations.

## Susceptibility of the bacterial isolates to plant extract

Agar well diffusion method was used to evaluate the antimicrobial activity of plants extracts. Similarly to the procedure used in disk-diffusion method, the agar plate surface was inoculated by spreading a volume of the microbial inoculum over the entire agar surface. Then, a hole with a diameter of 6 mm was punched aseptically with a sterile cork borer, and a volume (100 µL) of the extract solution at desired concentration was introduced into the wells. Then, the agar plates were incubated at 37ºC for 16 – 24 hours [40].

## Statistical data analysis

The data were analysed for statistical significance by one-way analysis of variance test using the Statistical Package of the Social Science (SPSS) version 16.0 (SPSS Inc., Chicago, IL, USA). Duncan’s multiple range test was applied to determine a significant difference (p≤0.05) if any, among the treatment groups.

# Results

## Prevalence of bacterial urinary tract infections among the study participants

**Table 1. and Figure 1. Prevalence of bacterial urinary tract infections among the study participants**

A total of 161 subjects presented with UTI were enrolled in the studies, out of the total samples examine, 32 samples yielded positive culture, while 129 have shown no growth. The prevalence rate of 19.9% was recorded in the study and the most implicating organisms causing urinary tract infections in the study were *Escherichia coli,* they were responsible for 41.0% of the cases of UTI From the study participant ,this is followed by *Klebsiella pneumoniae* (28%), *Staphylococcus aureus* (22%), and *Proteus mirabilis* (9%). The prevalence rate of 19.9% was recorded in this study ( Table 4.1)

Table 1. Distribution of bacterial isolates from the study participants

**S/No	Organisms	Frequency (n)	Percentage (%)**

**1**	*Escherichia coli*	13	40.6

**2**	*Klebsiella pneumoniae*	9	28.1

**3**	*Proteus mirabilis*	3	9.3

**4**	*Staphylococcus aureus*	7	21.8

**Total	32	100.0**

**KEY: n= Frequency, % = Percentage.** **The % prevalence rate of 19.9% was recorded in the study**

Figure 1. Prevalence of bacterial urinary tract infections among the study participants attending Aminu Kano Teaching Hospital Kano

## Distribution of bacterial isolates from urinary tract infected patients attending Aminu Kano Teaching Hospital Kano

The pie chart below shows the distribution of bacterial isolate that causes urinary tract infecton. The most implicating organisms causing urinary tract infections in the study were *Escherichia coli,* they were responsible for 41.0% of the cases of UTI From the study participant ,this is followed by *Klebsiella pneumoniae* (28%), *Staphylococcus aureus* (22%), and *Proteus mirabilis* (9%). The results are shown below in figure 2.

Figure 2. Distribution of bacterial isolates from urinary tract infected patients attending Aminu Kano Teaching Hospital Kano

## Antibacterial susceptibility testing of *Cassia tora* leaves extracts against the bacterial isolates

The bacterial isolates were screened for antibacterial activities using *Cassia tora* leaf extract in varying concentration and a conventional antibiotic (ciprofloxacin 5ug) were used as the controls. The convectional antibiotic shows sensitivity at 5ug, however the clinical isolates showed relatively high resistance to the plant extract (*Cassia tora*) at the concentration of 100mg/ml, 50mg/ml, 25mg/ml, 12.5mg/ml, 6.25mg/ml, 3.125mg/ml and 1.562mg/ml. The results are shown below in table 2.

## Minimum inhibitory concentration (MIC) and Minimum bactericidal concentration

The bacterial isolates were screened for antibacterial activities using *Cassia tora* leaf extract in varying concentration and a conventional antibiotic (ciprofloxacin 5ug) were used as the controls. The clinical isolates showed relatively high resistance to the plant extract (*Cassia tora*) at the concentration of 100mg/ml, 50mg/ml, 25mg/ml, 12.5mg/ml, 6.25mg/ml, 3.125mg/ml and 1.562mg/ml. The MIC was 0.00 mg/ml for all isolates tested as all tubes showed visible turbidity in the bacterial suspension containing *Cassia tora* leaves extracts.

Table 2. Antibacterial susceptibility testing of *Cassia tora* leaves extracts against the bacterial isolates

| BACTERIAL ISOLATES | CPFX(5ug)  S I R | | | 100mg/ml  S I R | | | 50mg/ml  S I R | | | 25mg/ml  S I R | | | 12.5mg/ml  S I R | | | 6.25mg/ml  S I R | | |
| --- | --- | --- | --- | --- | --- | --- | --- | --- | --- | --- | --- | --- | --- | --- | --- | --- | --- | --- |
| *E. coli* (n=13) | 10 | 3 | 0 | 0 | 0 | 13 | 0 | 0 | 13 | 0 | 0 | 13 | 0 | 0 | 13 | 0 | 0 | 13 |
| *K. pneumonia* (n=9) | 7 | 2 | 0 | 0 | 0 | 9 | 0 | 0 | 9 | 0 | 0 | 9 | 0 | 0 | 9 | 0 | 0 | 9 |
| *P. mirabilis* (n=3) | 2 | 1 | 0 | 0 | 0 | 3 | 0 | 0 | 3 | 0 | 0 | 3 | 0 | 0 | 3 | 0 | 0 | 3 |
| *S. aureus* (n=7) | 4 | 1 | 2 | 0 | 0 | 7 | 0 | 0 | 7 | 0 | 0 | 7 | 0 | 0 | 7 | 0 | 0 | 7 |
| Total | 23 | 7 | 2 | 0 | 0 | 32 | 0 | 0 | 32 | 0 | 0 | 32 | 0 | 0 | 32 | 0 | 0 | 32 |
| p- value |  | 0.04 |  |  | 1 |  |  | 1 |  |  | 1 |  |  | 1 |  |  | 1 |  |

P-value =1 the proportion of resistance are exactly equal

KEYS: S=Sensitive, I= Intermediate, R= Resistant, Ciprofloxacin= CPFX, *E. coli* = *Escherichia coli, k. pneumoniae= klebsiella pneumoniae, P.mirabilis= Proteus mirabilis, S.aureus= Staphylococcus aureus.*

Table 3. Minimum inhibitory concentration (MIC) and Minimum bactericidal concentration

| Test Organism | MIC (mg/ml) | MBC (mg/ml) |
| --- | --- | --- |
| *E. coli* | 0.00±0.00 | 0.00±0.00 |
| *K. pneumoniae* | 0.00±0.00 | 0.00±0.00 |
| *P. mirabilis* | 0.00±0.00 | 0.00±0.00 |
| *S. aureus* | 0.00±0.00 | 0.00±0.00 |
| p-value | 1 | 1 |

P-value =1 the proportion of resistance are exactly equal

Key: MIC= Minimum inhibitory concentration, MBC= Minimum bactericidal concentration

*E. coli* = *Escherichia coli, k. pneumoniae= klebsiella pneumoniae, P. mirabilis= Proteus mirabilis, S. aureus= Staphylococcus aureus*

## Distribution of UTI among the study participant based on gender and Age group

A total of 161 participant were enrolled in the studies out of which 92 were female with a percentage of 57.1% and 69 males with a percentage of 42.9% was recorded. The frequency of females presented with urinary tract infection (UTI) was more that of the males by difference of 23 (14%). Subjects with UTI infections ranged in age 4-15 to 49-60 with age group of 16-26 years being the highest number of participants presented with UTI. The distribution of cases of UTI according to gender (69 male, 92 females) and age group of pat participants is shown in table 4: and 5:

Table 4. Distribution of UTI among the study participants based on gender group

| **Genders** | **Frequency (n)** | **Percentage (%)** |
| --- | --- | --- |
| Male | 69 | 42.9 |
| Female | 92 | 57.1 |
| **Total** | **161** | **100.0** |

.

Table 5. Distribution of UTI among the study participants based on Age group

| **Age** | **Frequency (n)** | **Percentage (%)** |
| --- | --- | --- |
| 4-15 | 39 | 24.2 |
| 16-26 | 63 | 39.1 |
| 27-37 | 37 | 23.0 |
| 38-48 | 7 | 4.3 |
| 49-60 | 15 | 9.3 |
| **Total** | **161** | **100.0** |

Key: n=number, %= percentage

# Discussion

In this study, one hundred and sixty one (161) urine samples from patients attending Aminu Kano Teaching Hospital, Kano Nigeria were analyzed. Out of the total sample under study 32, (19.9%) samples yielded positive cultures, while 129 (80.1%) have shown no growth. The prevalence rate of 19.9% was recorded in this study. it was higher than the prevalence rate of 11.9% reported by [36]. It was also higher than 12.5% recorded by [41]. The increase maybe due to the occurrences of recurrent urinary tract infections, risk factors associated with UTI and multi-drug resistance pattern of the causative organism indicated in UTI.

The most implicating organisms causing urinary tract infections in the study were *Escherichia coli,* they were responsible for 41.0% of the cases of UTI. This was followed by *Klebsiella pneumoniae* (28.0%), *Staphylococcus aureus* (22%.0) and *proteus mirabili* (9.0%). These findings are similar to other reports which suggest that Gram-negative bacteria, particularly *Escherichia* *coli* is the commonest pathogen isolated in patients with UTI. *E. coli* was the most commonly isolated pathogen in significant bacteriuria. This same pattern was also reported by [42]. Women are more susceptible to UTI than men this is due to the nature of their urethra which is shorter than that of the men. UTIs are the key reason we’re often told to wipe from front to back after using the toilet. That’s because the urethra is located relatively close to the anus. Bacteria from the large intestine, such as *E. coli*, are in the perfect position to escape from the anus and invade the urethra. From there, they can travel up to the bladder, and if the infection isn’t treated, continue on to infect the kidneys. Infection of the bladder is the most common cause of cystitis-inflammation of the bladder. Women may be especially prone to UTIs because they have a shorter urethra, which allow bacteria quick access to the bladder. Women who use diaphragms for birth control may be at higher risk, as well as women who use spermicidal agents. After menopause, a decline in circulating estrogen causes changes in the urinary tract that make women more vulnerable to infection.

The bacterial isolates were screened for antibacterial activities using *Cassia tora* leaf extract in varying concentration and a conventional antibiotic (ciprofloxacin 5ug) were used as the controls. The clinical isolates showed relatively high resistance to the plant extract (*Cassia tora*) with a zone of inhibions 0 ± 2 mm at the concentration of 100mg/ml, 50mg/ml, 25mg/ml, 12.5mg/ml, 6.25mg/ml, 3.125mg/ml and 1.562mg/ml .This is in conformity with some of the findings reported by [43]. Whose findings reported that *Staphylococcus aureus* and *pseudomonas aeruginosa* displayed resistance in the in-vitro antibacterial susceptibility test but was sensitive to *Escherichia coli* indicating some differences in the result finding? The outcomes of the research also differ with that of [44]. Whose result showed antibacterial activity of ethanolic extract at (0.15mg/ml) and aqueous extract (0.31mg/ml) against various bacteria but maximum activity against *Staphylococcus aureus*, *lacto bacillus* and moderate activity against *pseudomonas aeruginosa*, *proteus vulgaris* and enterobacter with no activity against *Escherichia coli* and *salmonella typhi*. According to Clinical and laboratory standard institute-CLSI [45]. any plant material should be considered an effective therapeutic agent if it extract produces zones of inhibition of between 15-22 mm on the target pathogenic organism. Activity of plant extracts to test bacteria is normally expressed in-vitro as zones of inhibition in millimetre (greater than or equals to 7mm) around the organism on test [46]. The comparisons between the antibacterial activities of methanolic extracts of *Cassia tora* and that of standard antibiotics (Ciprofloxacin) showed that the extracts had an insignificant antibacterial activity when compare with the antibiotic. The antibiotic had better activity against the bacterial isolates. The better activity of the standard antibiotic over the extracts could be due to the fact that organic extracts are in crude and not in pure form as the synthetic antibiotics; hence the secondary active metabolites could be present in low concentration or even be masked in the extracts [47].

The tested bacteria showed different levels of susceptibility to the convectional antibiotics used in the study as shown by the zones of inhibition. The highest activity (30 mm) was recorded for ciprofloxacin against *Staphylococcus aureus,* followed by, *Escherichia coli* and *Proteus mirabilis*. Gram positive bacteria are often more susceptible to antibiotics than the gram negative bacteria due to the fact that gram negative organisms have an additional outer phospholipids membrane and porines that makes the cell wall impermeable to lipophilic and hydrophilic solutes respectively [48].

# Conclusion and Recommendation

The prevalence of UTI was determine to be 19.9% of which The most implicating organisms causing urinary tract infections in the studies were *Escherichia coli* responsible for 41.0% of the cases of UTI. This was followed by *K. pneumoniae* (28.0%), *P. mirabilis* (9.0%) and *S. aureus* (22%.0).

The clinical isolates tested showed resistance pattern to the methanolic extracts of *Cassia tora* leaves at different concentration. This indicates that, not all *Cassia tora* leaves contains significant amount of polyphenolic compound that can inhibit bacterial growth. This may be attributed to difference in geographical distribution of plant, effect of climates changes, genetic diversity and other factors.

## Recommendation

Base on the findings of this work, I hereby recommend the following

Further research should be carried out to quantify, compare and contrast between poly phenolic compounds of *Cassia tora* from different geographical distribution and to evaluate their similarities and difference in activities in order to avoid unnecessary consumption by the locals. Quantitative Chromatographic method for detection of phenolic component, antimicrobial susceptibility testing using different extraction solvent, Comparison of similarity and difference between *Cassia tora* plant extract from different geographical locations.

# Acknowledgment

Special gratitude to the experts for their constructive opinions and lecturers who assisted in writing the paper and designing experiments. As a result of this declaration and all authors agree to the publication of this study.

# Statement

There is no conflict of interest in this study. All authors agree to publish

# References

1. Amna, M. A., Chazan, B., Raz, R., Edelstein, H. and Colodner, R. (2013). Risk factors for non-Escherichia coli community-acquired bacteriuria Infection. *BioMed Central*, **41**: 473–477. <https://doi.org/10.1007/s15010-012-0347-1>
2. Foxman, B. (2014). Urinary Tract Infection Syndromes: occurrence, recurrence, bacteriology, risk factors, and disease burden*. Infectious Disease Clinics of North America,* **28**(1): *1–13.* doi:10.1016/j.idc.2013.09.003
3. Salvador, E., Wagenlehner, F. and Kohler, C. D. (2012). Comparison of asymptomatic bacteriuria Escherichia coli isolates from healthy individuals versus those from hospital patients shows that long-term bladder colonization selects for attenuated virulence phenotypes. *Infection and Immunity*, **80**: 668–78
4. Liza, D.L.T. and Jonathan, O. (2006). Urinary Tract Infections: Causes, Symptoms and Treatment. American *Journal of Epidemiology*, 132,328-337
5. Hooton, T. M. (2012). Uncomplicated urinary tract infection. *New England Journal of Medicine*, **366**(11): 1028-1037.
6. Gallagher, S. A. and Hemphill, R. R. (2001). Urinary TractInfections :Epidemiology ,detection and evaluation. <http://www.thrombosisconsult>. co./articles/textbooks. Accessed on 10/5/2023.
7. Suetens, C., Latour, K., Kärki, T., Ricchizzi, E., Kinross, P., Moro, et al (2018). Prevalence of healthcare-associated infections, estimated incidence and composite antimicrobial resistance index in acute care hospitals and long-term care facilities: results from two European point prevalence surveys, 2016 to 2017. *Euro surveillance : European communicable disease bulletin*, **23**(46): 1800516. <https://doi.org/10.2807/1560-7917.ES.2018.23.46.1800516>
8. Hudson, R. E., Job, K. M., Sayre, C. L., Krepkova, L. V., Sherwin, C. M. and Enioutina, E. Y. (2022). Examination of Complementary Medicine for Treating Urinary Tract Infections Among Pregnant Women Children.*Frontiersinpharmacology*,13**: 883216**. ://doi.org/10.3389/fphar.2022.883216
9. Flores-Mireles, A. L., Walker, J. N., Caparon, M. and Hultgren, S. J. (2015). Urinary tract infections: epidemiology, mechanisms of infection and treatment options. *Nature reviews Microbiology*, **13**(5): 269–284. <https://doi.org/10.1038/nrmicro3432>
10. Magill, S. S., O'Leary, E., Janelle, S. J., Thompson, D. L., Dumyati, G., Nadle, J., et al (2018). Changes in Prevalence of Health Care-Associated Infections in U.S. Hospitals. *The New England journal of medicine*, **379**(18): 1732–1744. <https://doi.org/10.1056/NEJMoa1801550>
11. Chedi, B. A. Z., Wannang, N. N., Halliru, M. A. and Bichi, L. A.(2009). A seven months retrospective study on urinary tract infection among patients at Aminu Kano Teaching Hospital. *Bayero journal of pure and Applied sciences*, **2**(2): 95-98.
12. Naber, K. G., Tirán-Saucedo, J., Wagenlehner, F. M. E. and RECAP group. (2022). Psychosocial burden of recurrent uncomplicated urinary tract infections. *GMS infectious diseases*, **10**: Doc01. <https://doi.org/10.3205/id000078>
13. Wullt, B., Sundén, F. and Grabe, M. (2019). Asymptomatic Bacteriuria is Harmless and Even Protective: Don't Treat if You Don't Have a Very Specific Reason. *European urology focus*, **5**(1): 15–16. <https://doi.org/10.1016/j.euf.2018.07.004>
14. Alao, F. O., Ololade, Z. S. and Nkeonye, C. V. (2018). Phytochemical and Antibacteria Potentials of Senna tora leaf and Seed extracts against Some Clinically Isolated Bacteria. *Journal of Bacteriology and Parasitology,* **9**(3): 2-4.
15. Ingle, A., Ranaware, P., Ladke, A. and Damle, M. (2012). *Cassia tora*: Phytochemical and pharmacological activity. *International Imperial Journal of Pharmacognosy and Natural Products*, **2**: 14-23.
16. Centers for Disease Control and Prevention (CDC). (2021). antibiotic-use. Accessed May 13, 2023. <https://www.cdc.gov/antibiotic-use/uti.html#anchor_1566489283094>
17. Hannan, T. J., Totsika, M., Mansfield, K. J., Moore, K. H., Schembri, M. A., and Hultgren, S. J. (2012). Host–pathogen checkpoints and population bottlenecks in persistent and intracellular uropathogenic Escherichia coli bladder infection. *FEMS microbiology reviews*, **36**(3): 616-648.
18. Komala, M., Bhowmik, D., and Kumar, K. P. (2013). Urinary tract infection: Causes, symptoms, diagnosis and it’s management. *Journal of Chemical and Pharmaceutical Sciences*, **6**: 22–28.
19. Meena, A. K., Niranjan, U. S., Yadav, A.K., Singh, B., and Nagariya, A.K. (2010). *Cassia tora* Linn: A review on its ethnobotany, phytochemical and pharmacological profile. *Journal of Pharmaceutical Research*, **3**: 557-560.
20. Yen, G. C., Chen, H. W. and Duh, P. D. (1998). Extraction and identification of an antioxidative component from Jue Ming Zi (*Cassia tora* L.). *Journal of Agricultural and Food Chemistry,* **46**: 820-824. DOI: 10.1021/jf970690 .
21. Poulsen, H.O., Johansson, A., Granholm, S., Kahlmeter, G. and Sundqvist, M. (2013). High genetic diversity of nitrofurantoin- or mecillinam-resistant Escherichia coli indicates low propensity for clonal spread. Journal of Antimicrobial Chemotherapy, **68**(9):1974–1977, <https://doi.org/10.1093/jac/dkt159>
22. Zhenbao, J., T. Fei, L. Guo, T. Gunjun and D. Xiaolin, 2007. Antioxidant properties of extracts from juemingzi (Cassia tora L.) evaluated in vitro. LWT-Food Sci. Technol., **40**: 1072-1077. DOI: 10.1016/j.lwt.2006.05.010
23. Zhu, L., Y. Shujuan, Z. Xinan, F. Xiong and Z. Mouming, 2008. Preparative separation and purification of five anthraquinones from Cassia tora L. by high-speed counter-current chromatography. Sep. Purif. Technol., **63**: 665-669.
24. Kirtikar, K. R. And Basu, B. D. (1975). Indian Medicinal Plants, Periodical Experts D- 42, VivekVihar Delhi, 2, 877.
25. Harshal, A.P. and Priscilla, M. D (2011). *CASSIA TORA* LINN. *International journal of pharmaceutical science and research*, **2**(9): 2286-2291
26. Kang, S.H., Lee, W.H., Lee, C.M., Sim, J.S., Won, S.Y., Han, S.R., et al (2020). Denovo transcriptome sequence of Senna tora provides insights into anthraquinone biosynthesis. *PLoSONE.* **15** (5). https://doi.org/10.1371/ journal.pone.0225564
27. Thakur, R. S., Puri, H. S. and Husain, A. (1989). Major Medicinal Plants of India, 1st edition, 143.
28. Roopashree, T. S. and Dang, R. (2017). Standardization and phytochemical investigation of Calendula officinalis, *Cassia tora* and Momordicaacharantia seed extract*. Journal of Pharmacy* *Research*, **16:** 85.
29. Hosseinzadeh, S., Jafarikukhdan, A., Hosseini, A. and Armand, R. (2015). The application of medicinal plants in traditional and modern medicine: A Review of Thymus vulgaris. *Internatinationl Journal of Clinical Medicine,* **6**: 635-642.
30. Randrianarivony, T. N., Ramarosandratana, A. V., Andriamihajarivo, T. H., Rakotoarivony, F. and Jeannoda, V. H. (2017). The most used medicinal plants by communities in Mahaboboka, Amboronabo, Mikoboka, South-Western Madagascar. *Journal of Ethnobiology and Ethnomedicine,* **13:** 1-12.
31. Falodun, A. (2010). Herbal medicine in Africa-distribution, standardization and prospects. *Research Journal of Phytochemistry,* **4**: 154-161.
32. Mafimisebi, T. E. and Oguntade, A. E. (2010). Preparation and use of plant medicines for farmers’ health in Southwest Nigeria: socio-cultural, magico-religious and economic aspects. *Journal of Ethnobiology and Ethnomedicine,* **6**: 1-9.
33. Cragg, G. M. and Newman, D. J. (2013). Natural Products: A Continuing Source of Novel Drug Leads. *Biochimica et Biophysica Acta,* **18**(30): 3670-3695
34. Atanasov, A. G., Waltenberger, B. P., ferschy-Wenzig, E., Linderd, T. and Wawrosch, C. (2015). Discovery and Resupply of Pharmacologically Active Plant Derived Natural Products: A Review*. Biotechnology Advances,* **33**: 1582-1614.
35. Barrister, A. I., Eme, A. and Okechukwu, I. (2015). Census politics in nigeria: an examination of 2006 population census. *journal of policy and development studies,* **9**(2): 157-9385. *Available in* [www.arabianjbmr.com/JPDS_index.php](http://www.arabianjbmr.com/JPDS_index.php).
36. Aiyegoro, O. A., Igbinosa, O. O., Ogunmwonyi, I. N., Odjadjare, E. E., igbinosa, O. E. and Okoh, A. I. (2007). Incidence of Urinary tract infections (UTI) among children and adolescents in lle-lfe, Nigeria. *African Medical Journal of Microbiology Research*, 013-019
37. Chesssbrough, (2006). District Laboratory practice in Tropical Countries. *Cambrigde University Press,* ***2****.* available in [www.cambrigde.org/9780521676311](http://www.cambrigde.org/9780521676311)
38. Cheesbrough M., (2009). District laboratory practice in tropical countries, second edition in New York in USA, chapter 7, (132-304).
39. Redfern, J., Kinninmonth, M., Burdass, D. and Verran, J. (2014). Using soxhlet ethanol extraction to produce and test plant material (essential oils) for their antimicrobial properties. *Journal of microbiology and biology education*, ***15***(1): 45–46. https://doi.org/10.1128/jmbe.v15i1.656
40. Balouiri, M., Sadiki, M. and Ibnsouda, S. K. (2016). Methods for in vitro evaluating antimicrobial activity. *Journal of pharmaceutical analysis,* **6**(2): 71-79
41. Taura, D.W. and Bashir, A. (2007). Bacterial agents of urinary tract infections and their current antibiotics sensitivity pattern among patients attending Aminu Kano Teaching Hospital (AKTH) kano, Nigeria. *International journal of biomedical and Health Sciences,* **3**(1*).*
42. Kolawole, A.S., Kolawole, O.M., Kandaki-olukemi, Y.T and Babantunse, S.K. (2009). Prevalence of Urinary tract infection and adolescent in ile-ife,nigeria. *African journal of Microbiology Research*, 1: 13-19.
43. Sahu, J., Koley, K.M. and Sahu, B.D. (2017). Attribution of antibacterial and antioxidant activity of *Cassia tora* extract toward its growth promoting effect in broiler birds. *Veterinary World*, **10**(2): 221-226.
44. Sharma, S., Dangi, M.S., Wadhwa, S., Daniel, V. and tiwari, A. (2010). Antibacterial Activity of *Cassia tora* Leaves. *International Journal of Pharmaceutical and Biological Archives*. **1**(1): 84 - 86.
45. Clinical Laboratory institute standards (CLSI), (2005). Performance standards for antimicrobial susceptibility 15^th^ informational supplement (CLSI document M100-S15). **25.**
46. Gislene, G.F., Locatelli, J., Freitas, P.C. and Silva, G.L. (2000). Antimicrobial activity of plant extracts and phytochemicals on antibiotic resistant bacteria. *Brazilian Journal of Microbiology*, **31** (04): 20-33.
47. Ahmed, I., Olonitola, O.S., Yakubu, S.E. and Ado, S.A. (2014). characterization and susceptibility of Escherichia coli isolated from infantile diarrhea to momordica charantia and Aeschynomene uniflora extracts in kano state, Nigeria. A PhD thesis, Ahmadu Bello University, zaria, Nigeria. *African journal of Microbiology Research,* 17-24.
48. Akortha, E.E. and Aluyi, H.S.A. (2002). Plasmid mediated antibiotic resistance among some members of the enterobacteraceae from diarrhoeal patients. *Tropical Journal of Environmental Science and Health*, **5**(1): 13 -16

# Supporting information

S1 Fig. This is the S1 Fig Title. Prevalence of bacterial urinary tract infections among the study participants attending Aminu Kano Teaching Hospital Kano

S2 Fig. This is the S2 Fig Title. Distribution of bacterial isolates from urinary tract infected patients attending Aminu Kano Teaching Hospital Kano

S1 Table. This is the S1 Table Title. Distribution of bacterial isolates from the study participants

S2 Table. This is the S2 Table Title. Antibacterial susceptibility testing of *Cassia tora* leaves extracts against the bacterial isolates

S3 Table. This is the S3 Table Title. Minimum inhibitory concentration (MIC) and Minimum bactericidal concentration

S4 Table. This is the S4 Table Title. Distribution of UTI among the study participants based on gender group

S5 Table. This is the S5 Table Title. Distribution of UTI among the study participants based on Age group

S1 File. This is the S1 File Title. Ethical approval letter

S2 File. This is the S2 File Title. Herbarium plant identification form

S3 File. This is the S4 File Title. work flowchart photos

# Abbreviations

(ASB): Asymtomatic bacteriuria

AKTH: Aminu Kano Teaching Hospital

*C. tora*: *Cassia tora*

CDC: Centers for Disease Control and Prevention

CLSI: Clinical and laboratory standards institute

CO2: Carbon dioxide

MIC: Minimum inhibitory concentration

TSI: Triple sugar iron agar

UTI : Urinary tract infection

W.H.O: World Health Organization
