# Supplementary material for: MaskDGNets: Masked-attention guided dynamic graph aggregation network for event extraction
Source: PLoS One. 2024 Nov 15;19(11):e0306673. doi: 10.1371/journal.pone.0306673 (PMC11567535; doi:10.1371/journal.pone.0306673)
Supplement: Attachment — Submitted filename: Respose_PONE-D-24-24636.docx [file pone.0306673.s002.docx]

Response to Reviewers

Dear Editors and Reviewers:

Thank you for giving us the opportunity to revise our paper. We have received your decision dated August 12, 2024, giving your revision requested on our paper entitled “**MaskDGNets: Masked-attention guided dynamic graph aggregation network for event extraction**” (**PONE-D-24-24636**). We have taken every effort to address the comments, which has enabled us to significantly improve the contributions and delivery of our paper.

We have made all the changes suggested by the reviewers and are herewith resubmitting the paper for further evaluation. The changes are highlighted in yellow color in the manuscript. The following are our responses to the specific points that required modification as the reviewers’ comments.

PLOS ONE Decision: Revision required [PONE-D-24-24636] - [EMID:5559582fb0e33f58]

PLOS ONE [em@editorialmanager.com](mailto:em@editorialmanager.com) Attachments Oct 9, 2024, 6:35 AM (4 days ago) to me

PONE-D-24-24636 MaskDGNets: Masked-attention guided dynamic graph aggregation network for event extraction PLOS ONE

Dear Dr. Zhang,

Thank you for submitting your manuscript to PLOS ONE. After careful consideration, we feel that it has merit but does not fully meet PLOS ONE’s publication criteria as it currently stands. Therefore, we invite you to submit a revised version of the manuscript that addresses the points raised during the review process.

Please submit your revised manuscript by Nov 22 2024 11:59PM. If you will need more time than this to complete your revisions, please reply to this message or contact the journal office at [plosone@plos.org](mailto:plosone@plos.org). When you're ready to submit your revision, log on to <https://www.editorialmanager.com/pone/> and select the 'Submissions Needing Revision' folder to locate your manuscript file.

Please include the following items when submitting your revised manuscript: A rebuttal letter that responds to each point raised by the academic editor and reviewer(s). You should upload this letter as a separate file labeled 'Response to Reviewers'. A marked-up copy of your manuscript that highlights changes made to the original version. You should upload this as a separate file labeled 'Revised Manuscript with Track Changes'. An unmarked version of your revised paper without tracked changes. You should upload this as a separate file labeled 'Manuscript'. If you would like to make changes to your financial disclosure, please include your updated statement in your cover letter. Guidelines for resubmitting your figure files are available below the reviewer comments at the end of this letter.

If applicable, we recommend that you deposit your laboratory protocols in protocols.io to enhance the reproducibility of your results. Protocols.io assigns your protocol its own identifier (DOI) so that it can be cited independently in the future. For instructions see: <https://journals.plos.org/plosone/s/submission-guidelines#loc-laboratory-protocols>. Additionally, PLOS ONE offers an option for publishing peer-reviewed Lab Protocol articles, which describe protocols hosted on protocols.io. Read more information on sharing protocols at <https://plos.org/protocols?utm_medium=editorial-email&utm_source=authorletters&utm_campaign=protocols>.

We look forward to receiving your revised manuscript. Kind regards,

Ghulam Mustafa, MS Computer Science Academic Editor PLOS ONE

**Response to Editor**

**Comment 1:**

When submitting your revision, we need you to address these additional requirements.

Please ensure that your manuscript meets PLOS ONE's style requirements, including those for file naming. The PLOS ONE style templates can be found at

<https://journals.plos.org/plosone/s/file?id=wjVg/PLOSOne_formatting_sample_main_body.pdf> and

<https://journals.plos.org/plosone/s/file?id=ba62/PLOSOne_formatting_sample_title_authors_affiliations.pdf>.

**Our Response to Comment 1:**

We are very grateful for the expert's constructive comments, and we have made detailed revisions based on them.

**Comment 2:**

Please note that PLOS ONE has specific guidelines on code sharing for submissions in which author-generated code underpins the findings in the manuscript. In these cases, all author-generated code must be made available without restrictions upon publication of the work. Please review our guidelines at <https://journals.plos.org/plosone/s/materials-and-software-sharing#loc-sharing-code> and ensure that your code is shared in a way that follows best practice and facilitates reproducibility and reuse.

**Our Response to Comment 2:**

We are very grateful for the expert's constructive comments, and we have made detailed revisions based on them. After the paper is officially published, we will organize and make the relevant codes public.

**Comment 2:**

Thank you for uploading your study's underlying data set. Unfortunately, the repository you have noted in your Data Availability statement does not qualify as an acceptable data repository according to PLOS's standards.

At this time, please upload the minimal data set necessary to replicate your study's findings to a stable, public repository (such as figshare or Dryad) and provide us with the relevant URLs, DOIs, or accession numbers that may be used to access these data. For a list of recommended repositories and additional information on PLOS standards for data deposition, please see <https://journals.plos.org/plosone/s/recommended-repositories>.

**Our Response to Comment 3:**

We are very grateful for the expert's constructive comments, and we have made detailed revisions based on them. For instance,

DuEE: https://figshare.com/articles/dataset/DuEE1_0_zip/27283248?file=49937835

CCKS2020:https://figshare.com/articles/dataset/ccks2020_zip/27283242?file=49937832

**Comment 4:**

Please review your reference list to ensure that it is complete and correct. If you have cited papers that have been retracted, please include the rationale for doing so in the manuscript text, or remove these references and replace them with relevant current references. Any changes to the reference list should be mentioned in the rebuttal letter that accompanies your revised manuscript. If you need to cite a retracted article, indicate the article’s retracted status in the References list and also include a citation and full reference for the retraction notice.

**Our Response to Comment 4:**

We are very grateful for the expert's constructive comments, and we have made detailed revisions based on them.

[8] Zhang P, Zheng J, Lin H, et al. Vehicle trajectory data mining for artificial intelligence and real-time traffic information extraction[J]. IEEE Transactions on Intelligent Transportation Systems, 2023, 24(11): 13088-13098.

[33] Li W, Cheng D, He L, et al. Joint event extraction based on hierarchical event schemas from framenet[J]. IEEE Access, 2019, 7: 25001-25015.

**Response to Reviewer 1#**

**Comment 1:**

Clarify the connection between the MaskDGNets framework and its components, such as MaskAM, DGAM, Bi-IndRNN+, and WVEM. Consider defining these acronyms upon first mention and providing a concise explanation of their individual roles.

**Our Response to Comment 1:**

We are very grateful for the expert's constructive comments, and we have made detailed revisions based on them. For instance,

The overall structure of the proposed MaskDGNets for event extraction framework is shown in Figure 1. The framework mainly consists of four parts: a word vector embedding module (WVEM), a sequence semantic extraction module (Bi-Indrnn+), a masked attention embedding module (MaskAM), and a dynamic graph aggregation module (DGAM). The word vector embedding module (WVEM) aims to obtain useful word vectors and character features and form interactive and complementary relationships. The sequence semantics extraction module (Bi-Indrnn+) mainly models the entire input sequence and extracts global and contextual semantics that benefit event representation while retaining local spatial features to the maximum extent. The mask attention embedding module (MaskAM) mainly improves the representation of events by fusing sequence features and word vector features. It uses a weight allocation strategy to highlight salient features and refine them at the same time. The dynamic graph aggregation module (DGAM) establishes dependencies between events and events and between events and primary attributes. It mines hidden correlations between them through dynamic node aggregation and transfer functions to improve the performance of event extraction.

**WVEM:** This module mainly consists of a word vector mapping layer (WVL) and a single-character embedding layer (CEL). The word vector mapping layer (WVL) mainly maps words in text or sentences to low-dimensional space to better represent word vector features. Single characters are essential components of words, and different characters have different semantics or contexts in words or phrases. To better learn word vector features and sentence representation, we map characters separately to reduce the semantics of the same character in different phrases, which helps to improve the representation of keywords in sentences.

**Bi-Indrnn+:** Recurrent neural networks (RNNs) have been proven effective in obtaining helpful sequence information for text or sentence sequence tasks. However, RNNs are prone to problems such as fitting and gradient explosion, and it is challenging to establish practical long-term dependencies. Compared with traditional RNNs, independent recurrent neural networks (Indrnn) can effectively solve the gradient vanishing and gradient explosion problems and thoroughly learn long-term dependencies because neurons are independent of each other and can transmit information across layers. At the same time, with the help of non-saturated activation functions such as ReLu, IndRNN becomes very robust after training, and by stacking multiple layers of IndRNN, a deeper network than the existing RNN can be constructed. Therefore, we designed an improved bidirectional independent recurrent neural network (Bi-Indrnn+), combining a set of $3\times3$ convolution and adaptive pooling operations with independent recurrent units. We squeezed the input feature information to reduce the number of calculation parameters and obtain the sequence details of sentences or texts.

**MaskAM:** Adequate contextual semantics help improve the representation of global features. It can increase the association between keywords and event types in sentences or documents, reduce the errors caused by the same entities or keywords in different event types, and better refine local details. The specific steps are as follows. The local features of the input sentence or text can effectively update the query features and collect context information by paying attention to each query. At the same time, these local features are refined.

**DGAM:** A graph convolutional neural network is a network that can perform convolution operations directly on topological graphs. At the same time, it can learn node representation by modeling the complex structure and rich semantics of the graph through nodes' aggregation and transmission capabilities. Therefore, to improve the description of event types or behaviors in sentences or texts and to better mine the hidden associations between events and events and between events and other attributes, we constructed a sizeable topological graph whose graph nodes are mainly two types of nodes, event types and essential attributes of events, to capture the associations between them, and further improve the representation of local features, global features, and contextual semantics, thereby improving the overall performance of the network.

**Comment 2:**

In the section comparing the MaskDGNets framework to state-of-the-art methods, the analysis could be enriched by discussing more about the underlying reasons why MaskDGNets outperforms the other methods, beyond the broad statement about graph aggregation and bi-directional RNNs. Discuss more on the architectural or design choices that may contribute to its success.

**Our Response to Comment 2:**

We are very grateful for the expert's constructive comments, and we have made detailed revisions based on them. For instance,

From Table 1, we can get the following conclusions.

(1) On the two baseline data sets of DuEE and CCKS2020, our proposed MaskDGNets event extraction framework achieved the optimal performance, and $F_{1}$ was 81.443% and 87.382%, respectively. The possible reason is that, on the one hand, the spatial and sequence details in the sentence are refined through the mask attention module, and the representation of salient features is highlighted by assigning different weights; at the same time, the improved two-way independence is adopted Recurrent neural network models contextual semantics from both positive and negative directions and strengthens long-term dependencies. On the other hand, the dynamic graph aggregation module alleviates the differences between hierarchical graph structures and attribute nodes. It enhances the interaction between events and related attribute information through encoding and dynamic aggregation, improving the overall framework performance.

In addition, single-character features are embedded into word vectors to avoid ambiguity in the exact character representation in different words. When word vector features are insufficient without adding auxiliary information, character features are used as the main features for representation, which is beneficial for describing event types in sentence sequences. At the same time, a new weighted loss function is used to perform separate supervised learning and optimization adjustments on each module so that the proposed MaskDGNets event extraction framework can learn the optimal features, thereby improving event extraction performance.

(2) Graph methods are more competitive on both datasets than traditional deep learning methods. On the DuEE dataset, EE-GCN of $F_{1}$ is 6.164% and 11.454% higher than PLMEE and JRNN, respectively. On the CCKS2020 dataset, GCN-ED of P is 6.849% and 10.18 higher than BERT-CRF and DMCNN, respectively. EE-GCN integrates syntactic structure and dependent label types and learns and updates graph representations context-dependently, thereby obtaining better extraction capabilities. The GCN-ED method pools the convolution vector of the current word and the entity information in the sentence while integrating syntactic information, thereby improving the extraction performance. In addition, on these datasets, EE-GCN and GCN-ED have higher $F_{1}$ values than Multi-GAT because the graph attention mechanism focuses on capturing the global details of events in the sentence while ignoring local details.

(3) On the DuEE dataset, compared with the Bi-LSTM and JMEE methods, the $F_{1}$ of PLMEE increased by 10.082% and 18.641, respectively, and the R-value of BERT-CRF increased by 6.975% and 12.813%, respectively. On the CCKS2020 dataset, the P and $F_{1}$ values of JRNN increased by 3.155% and 3.694%, respectively, compared with JMEE, and by 2.345% and 2.004%, respectively, compared with DMCNN. The possible reason is that the JRNN method fully uses the local and global structural information of events in sentences and reduces the error propagation from upstream components to downstream classifiers. At the same time, the interdependence between event triggers and argument roles is strengthened through global features, thereby improving the performance of event extraction.

**Comment 3:**

The absence of the actual figures mentioned (e.g., Figures 2 and 3) in the provided text makes it harder to follow the conclusions drawn. Ensure that the text clearly references these figures with interpretations that stand alone, even if the figures are not immediately visible.

**Our Response to Comment 3:**

We sincerely thank the reviewer for careful reading. As suggested by the reviewer, we have corrected these errors. For instance,

**Figure 1.** The overall network structure of MaskDGNets. Where, 'Bi-Indrnn+' indicates an improved bidirectional independent recurrent neural network module. 'WVL' indicates the word vector layers. 'CEL' indicates the character embedding layer. 'EM' indicates the embedding module. 'DGAM' indicates the dynamic graph aggregation module. 'MaskAM' indicates the mask attention module. indicates word vector feature with word and characters, the sentence sequence features and fusion feature by mask attention module. indicates the word feature and character feature. indicates the feature matrix multiplication. indicates the batch normalization operation. ${Weight}$ indicates the weight matrix. represents the recurrent unit. ${ReLu}$ represents the activation function. represents the adaptive pooling operation. indicates convolution operation. indicates the graph nodes, where indicates the event node in the sentence, represents the basic attribute node of the event in the sequence.

The overall structure of the proposed MaskDGNets for event extraction framework is shown in Figure 1. The framework mainly consists of four parts: a word vector embedding module (WVEM), a sequence semantic extraction module (Bi-Indrnn+), a masked attention embedding module (MaskAM), and a dynamic graph aggregation module (DGAM). The word vector embedding module (WVEM) aims to obtain useful word vectors and character features and form interactive and complementary relationships. The sequence semantics extraction module (Bi-Indrnn+) mainly models the entire input sequence and extracts global and contextual semantics that benefit event representation while retaining local spatial features to the maximum extent. The mask attention embedding module (MaskAM) mainly improves the representation of events by fusing sequence features and word vector features. It uses a weight allocation strategy to highlight salient features and refine them at the same time. The dynamic graph aggregation module (DGAM) establishes dependencies between events and events and between events and primary attributes. It mines hidden correlations between them through dynamic node aggregation and transfer functions to improve the performance of event extraction.

**Figure 2.** Validation loss of different event extraction methods.

According to Figure 2 clearly shows that the loss function of the MaskDGNets framework we proposed performs better in the training and verification phase. The descent process is relatively smooth, and at 50 iterations, its verification loss value is 0.09463, which is the lowest. This proves that the reconstruction loss function we designed plays a vital role in adjusting and optimizing the network and improving feature representation.

**Figure 3.** Efficiency of different event extraction methods. 'FLOPs' indicates Floating Point Operations Per Second. 'Params' indicates the parameter quantity.

According to Figure 3, the FLOPs of the proposed MaskDGNets event extraction framework are within an acceptable range, which is 9.8G. Compared with other event extraction methods, Bi-LSTM has the best performance in terms of time efficiency, which is 21.8G. Similarly, compared with the event extraction method based on topological graphs, the traditional event extraction method has a better competitive advantage. The possible reason is that the representation and calculation of graph node features are relatively complex, reducing the model's reasoning performance. The FLOPs of the BERT-CRF method are worse than those of Multi-GAT. It may be that the network structure of the BERT-CRF method is more profound, and the number of information transmission layers is greater, resulting in slow reasoning speed. In contrast, the Multi-GAT method mainly relies on data-driven to calculate graph node information, reducing the complexity of topological graph construction.

**Comment 4:**

In the discussion of the loss function, provide more context as to why certain loss functions outperform others. For example, if certain combinations of loss functions reduce class imbalance, explain this more fully. This can help reinforce the significance of the loss function choices and justify their inclusion.

**Our Response to Comment 4:**

We sincerely thank the reviewer for careful reading. As suggested by the reviewer, we have corrected these errors. For instance,

From Table 3, we can draw the following conclusions:

(1) The new weighted loss function we designed, through the primary loss function and multiple auxiliary loss functions acting together on the proposed MaskDGNets event extraction framework, can significantly improve the model's overall performance. For example, the $F_{1}$ of the $L_{a}+L_{b}+L_{c}+L_{m}$ method on the DuEE dataset is 0.569% and 0.891% higher than that of $L_{b}+L_{c}+L_{m}$ and $L_{a}+L_{b}+L_{m}$, respectively. This shows that the collaborative work of multiple loss functions can enable the network to obtain the optimal feature representation. At the same time, using a separate loss function to supervise each module can effectively suppress the class imbalance problem in the datasets. In particular, on the two baseline datasets, the $F_{1}$ ratio of $L_{b}+L_{c}+L_{m}$ is 0.322% and 0.434% higher than $L_{b}+L_{c}+L_{m}$, respectively. This also shows that using a separate loss function for supervised optimization of the dynamic graph aggregation module (DGAM) is beneficial to the aggregation and representation of graph node features, thereby improving the overall accuracy of the model.

(2) It can be seen from the two sets of datasets that the coordinated work of multiple loss functions is more effective than the supervised learning of the model using a single loss function. For example, on the CCKS2020 dataset, the ratio of and ${L_{c}}$ of the method is 0.295% and 0.429% higher than ${L_{m}}$, respectively. This shows that the coordinated work of multiple loss functions is beneficial to improving the model's overall performance. The of the method is 0.144% and 0.384% higher than that of the and methods, respectively. This shows that the primary loss function we designed plays an absolute leading role in the model's overall performance. It may be that taking the mean of the predicted probability can reduce the differences between the same category and further suppress the category imbalance problem in the datasets.

**Comment 5：**

Proof read the paper there are several grammatical errors.

**Our Response to Comment 5:**

We sincerely thank the reviewer for careful reading. As suggested by the reviewer, we have corrected these errors. For instance,

With the rapid development of the Internet and media, event extraction has been widely considered an essential information extraction task. Its primary purpose is obtaining all the information from complex, unstructured, or structured texts, sentences, and documents. All events included. Effective event extraction can significantly support the implementation of many downstream tasks, such as knowledge graph construction, social event detection, event prediction, and intelligence and business economic formulation. Then, as information grows exponentially, it will be difficult to fully capture the hidden correlations between events using simple machine learning or deep learning methods. Therefore, how to better capture the deep local sum of events in texts or sentences Global semantics and relevant contextual details to improve these correlations in events have become a research hotspot.

To address these limitations, many researchers have applied traditional deep learning technology to this task, relying on the powerful self-learning ability of neurons in the network and the ability to obtain deep discriminant features while reducing manual participation in feature screening and design and improving the performance of event extraction. For example, using a deep convolutional neural network to extract local spatial details of events in texts or sentences and using a recursive neural network to model global and contextual information, but due to the limitation of the receptive domain, it is difficult to capture the details of event attributes fully. Neurons in recurrent neural networks (RNNs) must improve their interpretability. At the same time, the network is prone to problems such as fitting and gradient explosion and cannot effectively establish long-term dependencies. There is still a lot of room for improvement in event extraction performance. The Long-short term memory networks (LSTMs) effectively alleviate the problems of RNNs and can describe text events from both positive and negative directions. Subsequently, some researchers considered the relationship between events and essential attributes such as entities, roles, and time. Graph methods, transformers, and joint extraction methods were widely used. Graph methods mainly establish connections between events and these essential attributes and achieve accurate descriptions of events through the convergence and transmission functions between nodes. Although these extraction methods using graph networks have achieved encouraging results, only the importance of local features is considered in constructing topological maps. In contrast, the importance of global information, contextual semantics, and the ambiguity and correlation between the same entity or other attribute information in different events are ignored.

Secondly, this feature information is processed and refined, the representation of salient features is highlighted, and redundant information is reduced. In addition, dependencies are established between events and essential attributes, thereby improving the performance of event extraction by mining hidden correlations between them.

This section first gives a basic overview of the proposed MaskDGNets event extraction framework and briefly introduces each module of internal structure, working principle, and training steps.

Reviewer #2: The manuscript was interesting and good written and designed. Introduction was good written Design of the study good. The illustrated figures were good and clear Discussion was good and including the conclusion of the study.

**Response to Reviewer 2#**

**Comment 1:**

The manuscript was interesting and good written and designed.
Introduction was good written
Design of the study good.
The illustrated figures were good and clear
Discussion was good and including the conclusion of the study.

**Our Response to Comment 1:**

Thank you for your letter and for the reviewers’ comments concerning our manuscript entitled “**MaskDGNets: Masked-attention guided dynamic graph aggregation network for event extraction**” (**PONE-D-24-24636**).Those comments are all valuable and very helpful for revising and improving our paper, as well as the important guiding significance to our researches. Special thanks to you for your good comments.
